# Structural, luminescence and thermometric properties of nanocrystalline YVO_4_:Dy^3+^ temperature and concentration series

**DOI:** 10.1038/s41598-019-38774-6

**Published:** 2019-02-14

**Authors:** I. E. Kolesnikov, A. A. Kalinichev, M. A. Kurochkin, E. V. Golyeva, A. S. Terentyeva, E. Yu. Kolesnikov, E. Lähderanta

**Affiliations:** 1 St. Petersburg State University, 7/9 Universitetskaya nab., 199034 St. Petersburg, Russia; 2 Lappeenranta University of Technology LUT, Skinnarilankatu 34, 53850 Lappeenranta, Finland; 3 Peter the Great St. Petersburg Polytechnic University, Polytechnicheskaya 29, 195251 St. Petersburg, Russia; 4 Scientific and Technological Institute of Optical Material Science, VNTs S. I. Vavilov State Optical Institute, Babushkina 36-1, 192171 St. Petersburg, Russia; 5 Volga State University of Technology, Lenin sqr. 3, 424000 Yoshkar-Ola, Russia

## Abstract

We report systematic study of Dy^3+^-doped YVO_4_ nanophosphors synthesized via modified Pechini technique. Effect of calcination temperature and doping concentration on structure and luminescence has been investigated. XRD and Raman spectroscopy revealed preparation of single phase nanoparticles without any impurities. Synthesized nanopowders consisted of weakly agglomerated nanoparticles with average size about 50 nm. Photoluminescence spectra of YVO_4_:Dy^3+^ nanoparticles consisted of the characteristic narrow lines attributed to the intra-configurational 4f-4f transitions dominating by the hypersensitive ^4^F_9/2_–^6^H_13/2_ transition. The calcination temperature variation did not affect ^4^F_9/2_ lifetime, whereas increase of doping concentration resulted in its gradual decline. Potential application of YVO_4_:Dy^3+^ 1 at.% and 2 at.% nanopowders as ratiometric luminescence thermometers within 298–673 K temperature range was tested. The main performances of thermometer including absolute and relative thermal sensitivities and temperature uncertainty were calculated. The maximum relative thermal sensitivity was determined to be 1.8% K^−1^@298 K, whereas the minimum temperature uncertainty was 2 K.

## Introduction

In last decades rare earth materials have been widely used as high-performance luminescent devices, catalysts, and other functional materials based on the electronic, optical, and chemical characteristics arising from their 4f electrons^1–5^. The unique 4f electronic configuration of rare earth elements makes them perfect phosphors emitting light ranging from UV to NIR. Hence, rare earth doped materials are potential candidates for design of multicolor LEDs^6^. Among rare earth ions, the dysprosium has attracted much attention due to its white light emission^7,8^. Dy^3+^ ions emit several luminescence bands in blue, green, yellow and red ranges of the spectrum, which intensities depend on the host environment. As a host for Dy^3+^ doping, various matrices have been extensively studied such as phosphates^9–11^, tungstate^12^, vanadate^13^, molybdate^14^ niobate^15^, silicates^16–18^, aluminate^19^, and borates^20,21^. Orthovanadates have many exceptional characteristics including excellent thermal, mechanical, and optical properties; which play an important role in many optical devices, catalysts, and laser host materials^22–24^. In particular, yttrium vanadate (YVO_4_) is very significant oxide in materials science and technology, for example, its large single crystal has been extensively used as an excellent polarizer and laser host material, whereas its powder doped with several rare earth ions has been used as an attractive phosphor due to its high luminescence quantum yield^25–27^. The previous works mainly focused on the Eu^3+^-doped YVO_4_ with different synthetic methods^28–30^, because it is a commercial red-emitting phosphor used in color television, the high-pressure mercury lamp, and as a scintillator in medical image detectors^31–34^. To the best of our knowledge, the systematic study of calcination temperature and doping concentration effect on properties of YVO_4_:Dy^3+^ nanophosphors has not been reported so far.

Recently, great attention was attracted to developing of non-contact luminescence thermometers with submicrometric spatial resolution. One of the most promising thermal sensing techniques is based on luminescence intensity ratio between emission bands arising from two thermally coupled levels. These levels should be closely spaced (the energy gap generally ranges from 200 to 2000 cm^−1^) and it is assumed to be in the thermodynamic quasi-equilibrium^35^. The main advantage of the ratiometric approach for temperature sensing is independence on spectrum losses and fluctuations of the excitation intensity, which leads to a much higher accuracy^36,37^. Emission bands of Dy^3+^ ions were originated from two thermally coupled levels (^4^I_15/2_ and ^4^F_9/2_) with the energy difference around 1000 cm^−1^. So, Dy^3+^-doped nanoparticles is suitable for temperature sensing with high spatial resolution and good sensitivity, which can be used in organisms or in living cells^36,38,39^.

This paper is focused on the detailed study of calcination temperature and doping concentration effect on the structure, steady-state and kinetics luminescence properties of YVO_4_:Dy^3+^ nanoparticles. Influence of excitation mechanisms on optimum doping concentration and quenching was explored. Synthesized samples were successfully used as ratiometric thermal sensors in wide temperature range of 298–673 K. The thermometric performances including thermal sensitivities, temperature uncertainty and repeatability were obtained.

## Results and Discussion

**Figure 1:**
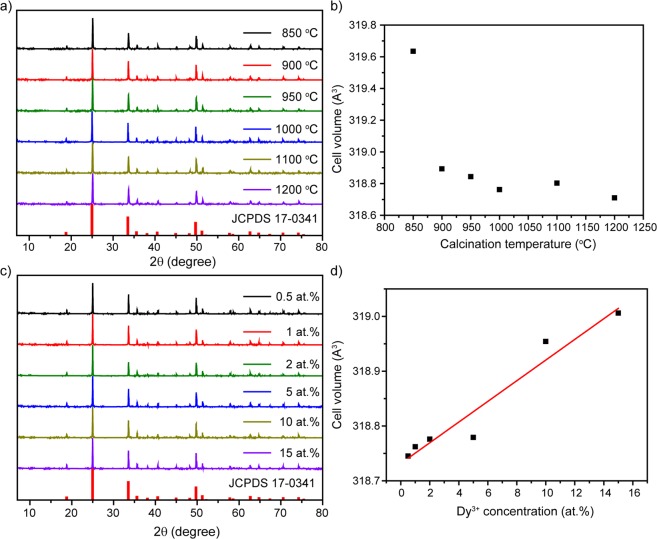
(**a**) XRD patterns of YVO_4_:Dy^3+^ 1 at.% temperature series and the standard card YVO_4_; (**b**) unit cell volume as a function of calcination temperature; (**c**) XRD patterns of YVO_4_:Dy^3+^ 1000 °C concentration series and the standard card YVO_4_; (**d**) unit cell volume as a function of Dy^3+^-doping.

Figure 1a shows XRD patterns of YVO_4_:Dy^3+^ 1 at.% samples prepared at different calcination temperatures. All diffraction peaks of powders can be indexed by the reference standard YVO_4_ (tetragonal phase, space group I41/amd, JCPDS 17-0341). No other crystalline phase was detected. The cell parameters were calculated with UnitCell software. As it can be seen from Fig. 1b, unit cell volume decreases along with increase of calcination temperature. Such behavior can be elucidated by formation of better crystalline structure of nanoparticles with cell volume closer to the monocrystal^40^.

Effect of Dy^3+^ doping concentration on YVO_4_ nanocrystalline powders is presented in Fig. 1c. XRD patterns confirm the presence of pure tetragonal phase without any structural impurities. Dysprosium ions substitute yttrium ions in D_2d_ site symmetry. The single-crystal cell volume systematically increases with growth of dopant concentration due to the difference of ionic radii between yttrium (r = 0.893 Å) and dysprosium ions (r = 1.03 Å). Noteworthy, the unit cell volume has a linear relationship with the amount of Dy^3+^, which is consistent with Vegard’s law (Fig. 1d). This result demonstrates that the dysprosium ion has been efficiently and homogeneously incorporated into the host matrix of YVO_4_ due to the similar ionic radius and chemical reactivity of Dy^3+^ and Y^3+ ^^41^.

**Figure 2:**
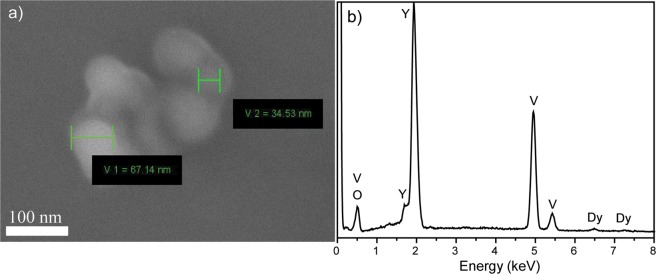
(**a**) SEM images of YVO_4_:Dy^3+^ 1 at.% 1000 °C nanopowder; (**b**) EDX analysis of YVO_4_:Dy^3+^ 1 at.% 1000 °C nanopowder.

Figure 2a shows scanning electron microphotograph of the synthesized YVO_4_:Dy^3+^ 1 at.% 1000 °C nanopowder. As seen from the micrograph, the powder consists of weakly agglomerated nanoparticles with average size about 50 nm. Elemental analysis was studied using EDX technique. We observed signals from yttrium, vanadium, oxygen, and dysprosium (Fig. 2b).

**Figure 3:**
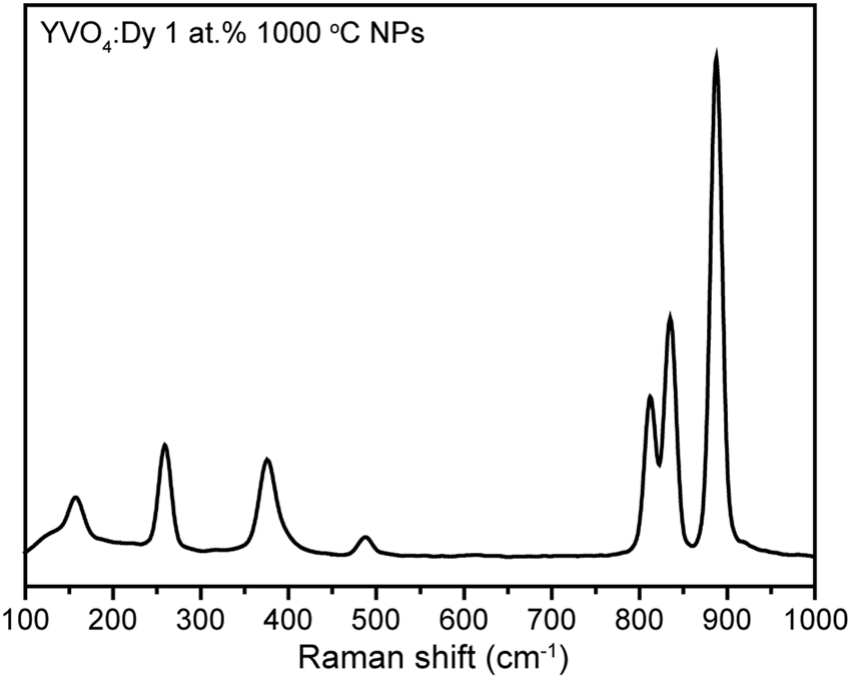
Raman spectrum of YVO_4_:Dy^3+^ 1 at.% 1000 °C nanopowder.

Structural properties of Dy^3+^-doped YVO_4_ nanopowders were studied also using vibrational spectroscopy. Figure 3 presents Raman spectrum of YVO_4_:Dy^3+^ 1 at.% 1000 °C measured in spectroscopic range from 100 to 1000 cm^−1^. As can be seen, this spectrum consists of several sharp Raman lines corresponding to the internal vibrations of VO_4_^3−^ group and external vibrations of VO_4_^3−^ tetrahedra and Y^3+^ ions in YVO4 unit cell^42^. The external vibration at 157 cm^−1^ (B_1g_(1)) can be attributed to the O–Y–O bending mode. The internal vibrations, which can be ascribed to the O–V–O bending and VO_4_ stretching modes, are located at higher frequencies: 259 (B_2g_), 376 (A_1g_(1)), 488 (B_1g_(3)), 812 (B_1g_(4)), 835 (E_g_(5)) and 888 cm^−1^ (A_1g_(2))^43^. The observed spectrum is dominated by the totally symmetrical vibration of VO_4_^3−^ tetrahedron^32^. Narrow width of Raman lines indicates good crystallinity and homogeneity of synthesized powder.

**Figure 4:**
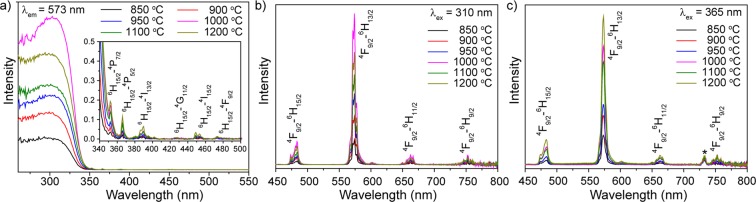
(**a**) Excitation spectra of YVO_4_:Dy^3+^ 1 at.% nanopowders calcinated at different temperatures (λ_em_ = 573 nm). Emission spectra of YVO_4_:Dy^3+^ 1 at.% nanopowders calcinated at different temperatures upon (**b**) λ_ex_ = 310 nm and (**c**) λ_ex_ = 365 nm excitation. The asterisk indicates the second order of excitation wavelength.

Further investigations were devoted to the influence of synthesis conditions (calcination temperature and doping concentration) on luminescence properties. Figure 4a shows excitation spectra within spectral range of 260–560 nm (λ_em_ = 573 nm) for nanocrystalline powders YVO_4_:Dy^3+^ 1 at.% calcinated at different temperatures. The obtained spectra consist of broad and intense band in the UV region and several narrow lines in visible region. The broad band can be assigned to the charge transfer from the oxygen ligands to the central vanadium atom inside the VO_4_^3−^ groups^44^. From the viewpoint of molecular orbital theory, it corresponds to transitions from the ^1^A_2_ (^1^T_1_) ground state to ^1^A_1_ (^1^E) and ^1^E (^1^T_2_) excited states of the VO_4_^3−^ ion^45^. Low peaks in visible region at longer wavelength are assigned to the intra-configurational 4f–4f transitions in dysprosium ions. The observed bands are attributed to the following transitions: ^6^H_15/2_–^4^P_7/2_ (352 nm), ^6^H_15/2_–^4^P_5/2_ (365 nm), ^6^H_15/2_–^4^I_13/2_ (387 and 390 nm), ^6^H_15/2_–^4^G_11/2_ (427 nm), ^6^H_15/2_–^4^I_15/2_ (449 and 453 nm) and ^6^H_15/2_–^4^F_9/2_ (473 nm).

Emission spectra of nanocrystalline powders YVO_4_:Dy^3+^ 1 at.% calcinated at different temperatures are shown in Fig. 4b,c. The measurements were conducted in the spectral range of 450–800 nm upon λ_ex_ = 310 nm and 365 nm radiation, which presents various excitation mechanisms. The emission spectra are dominated by green-yellow band (573 nm) corresponding to the hypersensitive ^4^F_9/2_–^6^H_13/2_ transition. Other observed lines are attributed to the ^4^I_15/2_–^6^H_15/2_ (455 nm), ^4^F_9/2_–^6^H_15/2_ (484 nm), ^4^F_9/2_–^6^H_11/2_ (662 nm), ^4^F_9/2_–^6^H_9/2_ (752 nm) transitions. ^4^F_9/2_–^6^H_13/2_ is the forced electric dipole transition, which is hypersensitive and its intensity can vary by orders of magnitude depending on the local site symmetry, whereas ^4^F_9/2_–^6^H_15/2_ transition intensity is insignificantly affected by the environment^46,47^.

**Figure 5:**
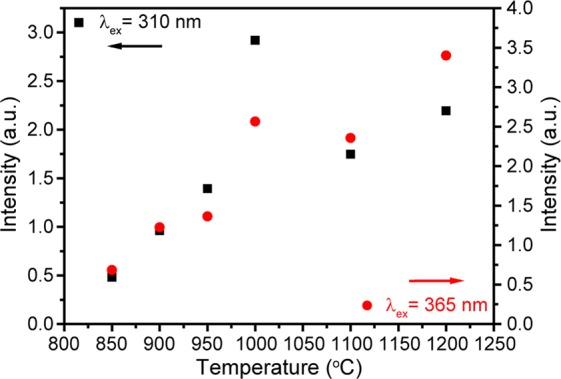
Dependence of ^4^F_9/2_–^6^H_13/2_ integrated intensity of YVO_4_:Dy^3+^ 1 at.% nanopowders on calcination temperature.

Integrated intensity of the most prominent ^4^F_9/2_–^6^H_13/2_ transition upon λ_ex_ = 310 and 365 nm radiation versus calcination temperature is shown in Fig. 5. As one can see, increase of calcination temperature leads to the growth of emission intensity, which coincides with usual behavior of rare earth-doped nanomaterials^48–50^. The observed growth is explained by an improvement of the sample crystallinity and a decrease of the number of OH^–^ groups on the surface of nanoparticles^51,52^. In addition, it is well known that a growth of the calcination temperature leads to an increase of nanoparticles size, which in turn also results in luminescence intensity enhancement due to reduction of doping ions fraction on the surface^53^.

**Figure 6:**
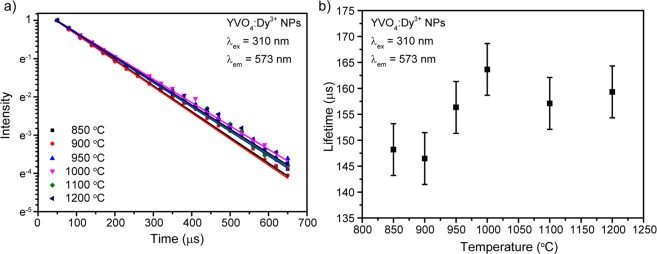
**(a)** Decay curves of YVO_4_:Dy^3+^ 1 at.% temperature series; **(b)** ^4^F_9/2_ lifetime as a function of calcination temperature.

To get more information about photoluminescence characteristics of prepared nanophosphors, fluorescence kinetics measurements were carried out. The photoluminescence decays of YVO_4_:Dy^3+^ temperature series were monitored at ^4^F_9/2_–^6^H_13/2_ transition upon 310 nm excitation (Fig. 6a). All experimental curves were fitted by single exponential function:

1 $$I={I}_{0}\cdot {e}^{-\frac{t}{{\tau }_{f}}}$$

where τ_f_ is the observed lifetime of ^4^F_9/2_ level. Figure 6b shows measured lifetimes dependence on calcination temperature. It should be noted that the increase of calcination temperature insignificantly affects the ^4^F_9/2_ lifetime.

**Figure 7:**
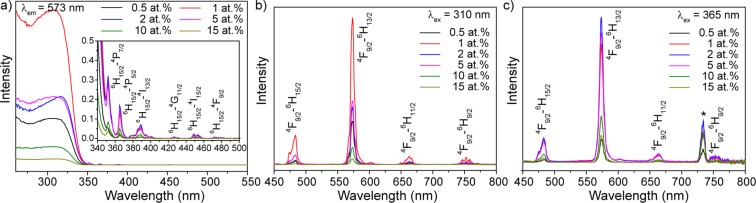
(**a**) Excitation spectra of YVO_4_:Dy^3+^ 1000 °C nanopowders with different doping concentrations (λ_em_ = 573 nm). Emission spectra of YVO_4_:Dy^3+^ 1000 °C nanopowders with different doping concentrations upon (**b**) λ_ex_ = 310 nm and (**c**) λ_ex_ = 365 nm excitation. The asterisk indicates the second order of excitation wavelength.

Next we discuss the effect of doping concentration on the luminescence properties of nanocrystalline powders YVO_4_:Dy^3+^. All samples of concentration series (1–15 at.%) were synthesized with calcination temperature of 1000 °C. Excitation spectra of nanocrystalline powders YVO_4_:Dy^3+^ 1000 °C with different doping concentrations are shown in Fig. 7a. These spectra consist of intense band attributed to the absorption of VO_4_^3−^ groups and weak Dy^3+^ 4f–4f transitions as it was described for temperature series. The spectral line positions of concentration series coincide with the temperature series. Emission spectra of nanocrystalline powders YVO_4_:Dy^3+^ 1000 °C with different doping concentrations upon 310 nm and 365 nm radiation are presented in Fig. 7b,c. The most intensive line in the measured spectra is attributed to the ^4^F_9/2_–^6^H_13/2_ transition.

**Figure 8:**
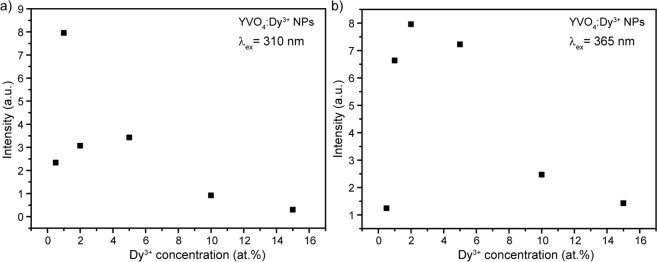
Dependence of ^4^F_9/2_–^6^H_13/2_ integrated intensity of YVO_4_:Dy^3+^ nanopowders on doping concentration upon **(a)** 310 nm and **(b)** 365 nm excitation.

Figure 8 presents the integrated luminescence intensity of the most intensive transition ^4^F_9/2_–^6^H_13/2_ as a function of doping concentration upon excitation wavelengths 310 and 365 nm. It is well-known that the optimum of Dy^3+^ concentration is determined by two competitive effects: on the one hand, an increase of the doping concentration means an increase of luminescence centers number and thus radiative recombination. On the other hand, there is also an increase of the probability of energy transfer between dysprosium ions, which enhances the efficiency of the nonradiative processes^54^. As can be seen, first emission intensity goes up with increasing doping concentration but further growth of Dy^3+^ ions number leads to intensity reduction. This indicates the concentration quenching effects for the luminescence of Dy^3+^ in YVO_4_ host. Noteworthy, the optimum doping concentration was found to be different for various excitation mechanisms. The optimum Dy^3+^ concentration for host excitation (310 nm) was determined to be 1 at.%, whereas for the direct Dy^3+^ ions excitation (365 nm) optimum concentration was 2 at.%. Similar difference in optimum concentration depending on excitation mechanism was previously observed for Eu^3+^ and Nd^3+^-doped YVO_4_ nanopowders^32,55^.

**Figure 9:**
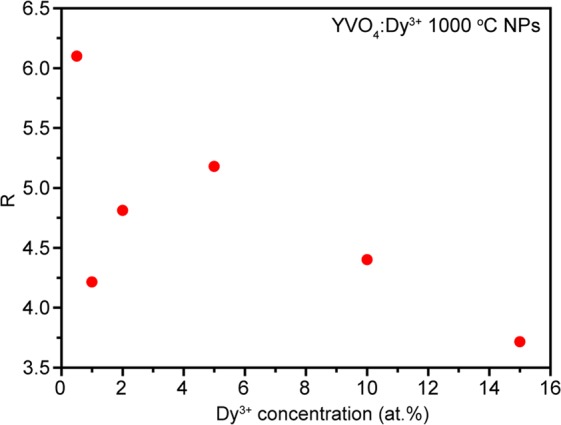
^4^F_9/2_–^6^H_13/2_ / ^4^F_9/2_–^6^H_15/2_ intensity ratio of YVO_4_:Dy^3+^ samples as a function of doping concentration.

To obtain information about Dy^3+^ concentration effect on the crystal structure and crystal field, ratio between ^4^F_9/2_–^6^H_13/2_ and ^4^F_9/2_–^6^H_15/2_ intensities (*R*) was calculated. This parameter is similar to the well-known asymmetry ratio for Eu^3+^ ions^56,57^. *R* value give information about the local surrounding and environmental changes near the Dy^3+^ ions. The higher the calculated parameter is, the more apart from a centrosymmetric geometry luminescent center is located. It is well-known that if Dy^3+^ is located at low symmetry without the inversion symmetry, the yellow emission is the most intense of all the transitions, as is the case with our synthesized nanocrystalline phosphors^58^. Figure 9 shows the dependence of *R* value, calculated from emission spectra obtained upon 365 nm excitation, on Dy^3+^ doping concentration for YVO_4_ host. As can be seen, the calculated ratio demonstrates nonmonotonical behavior, but main trend is decreasing of *R* value along with increasing of doping concentration. So, we can conclude that introduction of additional Dy^3+^ ions in YVO_4_ host results in growth of local symmetry.

**Figure 10:**
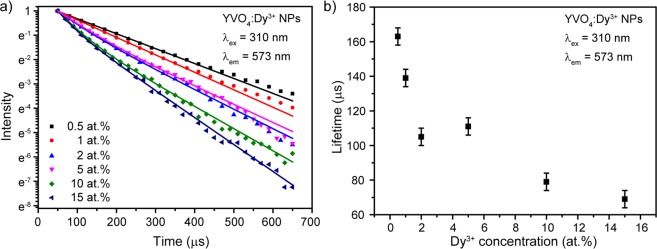
(**a**) Decay curves of YVO_4_:Dy^3+^ concentration series; (**b**) ^4^F_9/2_ level lifetime as a function of doping concentration.

Luminescence decays of YVO_4_:Dy^3+^ concentration series monitored at ^4^F_9/2_–^6^H_13/2_ transition upon 310 nm excitation are presented in Fig. 10a. Experimental data of synthesized nanocrystalline powders doped with 0.5 and 1 at.% were approximated by mono-exponential function. Higher doped samples demonstrate non mono-exponential decay, therefore, to provide correct fitting we used biexponential model. The average lifetime was obtained according following formula^59^:

2 $${\tau }_{av}=\frac{{A}_{1}{\tau }_{1}^{2}+{A}_{2}{\tau }_{2}^{2}}{{A}_{1}{\tau }_{1}+{A}_{2}{\tau }_{2}}$$

where A_1_ and A_2_ are pre-exponential factors; τ_1_ and τ_2_ are lifetimes. The fitting model was changed due to growth of Dy^3+^ doping ions located on the nanopartilces surface^60^. The non mono-exponential behavior is originated from the different decay rates of the Dy^3+^ ions situated at the surface and in the volume of the nanoparticles^61–63^. Figure 10b shows dependence of the obtained lifetimes on Dy^3+^ doping concentration. One can see that the lifetime gradually declines from 163 μs to 69 μs along with increase of Dy^3+^ ions number.

As it was previously observed, growth of the amount of Dy^3+^ ions in YVO_4_ host leads to the concentration quenching. According to Blasse *et al*., the energy transfer mechanism is defined by the critical energy transfer distance (*R*_*c*_) if the doping ions occupy single crystallographic position. The critical energy transfer distance was calculated by the following equation^64^:

3 $${R}_{c}\approx 2{[\frac{3V}{4\pi {x}_{c}N}]}^{\frac{1}{3}}$$

where *x*_c_ is the critical doping content (*x*_c_ = 0.01 or 0.02), *N* is the number of cation sites in the unit cell (*N* = 4 for YVO_4_), and *V* is the volume of the unit cell (*V* ≈ 318.78 Å^3^). Using aforementioned parameters, R_c_ was found to be 24.8 and 19.6 Å for host and direct excitations, respectively. Upon both excitation wavelengths calculated value of R_c_ is bigger than 5 Å, so exchange interaction cannot control energy transfer between Dy^3+^ ions in YVO_4_ host^65^. Therefore, we can conclude that the concentration quenching is caused by the multipolar interaction mechanism^66,67^. Multipolar interaction includes dipole–dipole (d–d), dipole–quadrupole (d–q), and quadrupole–quadrupole (q–q) interaction. An interaction type can be determined using formula proposed by Van Uitert^68^. Further, Ozawa and Jaffe modified it as follows^69^:

4 $$\frac{I}{x}=k{[1+\beta {(x)}^{\frac{\theta }{3}}]}^{-1}$$

where I is the integral intensity, x is the activator concentration, k and β are constant for the same excitation conditions for a given host crystal. According to the above equation θ = 3 for the energy transfer among the nearest neighbor ions, while θ = 6, 8 and 10 for d–d, d–q and q–q interactions, respectively^70,71^.

**Figure 11:**
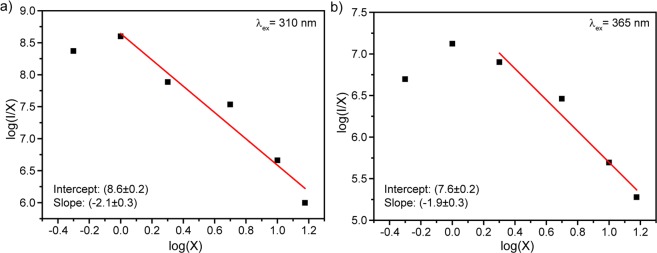
Logarithmic plot of YVO_4_:Dy^3+^ nanophosphors emission intensity vs doping concentration upon (**a**) 310 nm and (**b**) 365 nm excitation. Lines are a linear fit.

The critical concentration of Dy^3+^ ions was determined as 1 and 2% for 310 and 365 nm excitation, respectively. The dependence of the emission intensity of YVO_4_:Dy^3+^ nanopowders on the doping concentration was investigated. The multipolar character (θ) can be obtained by plotting log (I/x) vs log (x) as presented in Fig. 11. The slope θ/3 from approximation was determined to be −2.06 and −1.89 for 310 and 365 nm excitation, giving calculated value of θ is close to 6 in both cases. Therefore, concentration quenching in YVO_4_:Dy^3+^ nanocrystalline powders occurred due to dipole–dipole interaction regardless of the excitation mechanism.

In order to study the emission color dependence of the synthesized nanophosphors on calcination temperature and doping concentration, the Commission Internationale de L’Eclairage (CIE) chromaticity coordinates were calculated and the results are presented in Fig. S1 and Table 1. The chromaticity coordinates were obtained using emission spectra measured upon 310 nm excitation. As can be seen, both calcination temperature and doping concentration do not strongly influence on the emission color of Dy^3+^-doped YVO_4_ phosphor. Moreover, it should be noted that the observed small change of CIE coordinates has nonmonotonic behavior along with growth of calcination temperature or Dy^3+^ concentration.

**Table 1:** CIE coordinates of YVO_4_:Dy^3+^ temperature and concentration series.

Sample	CIE chromaticity coordinates	
	x	y
**Temperature series**		
850 °C	0.440	0.486
900 °C	0.439	0.486
950 °C	0.444	0.490
1000 °C	0.438	0.485
1100 °C	0.441	0.487
1200 °C	0.435	0.483
**Concentration series**		
0.5 at.%	0.449	0.498
1 at.%	0.425	0.474
2 at.%	0.433	0.480
5 at.%	0.436	0.485
10 at.%	0.431	0.479
15 at.%	0.444	0.490

During last decade a lot of attention is attracted to the search of convenient non-contact luminescence thermometer^72–74^. Due to the unique properties of rare earth ions, plenty organic and inorganic materials doped with lanthanides has been suggested for luminescence thermometry^75–77^. Here, we studied possibility to define local temperature using Dy^3+^-doped YVO_4_ nanophosphors.

**Figure 12:**
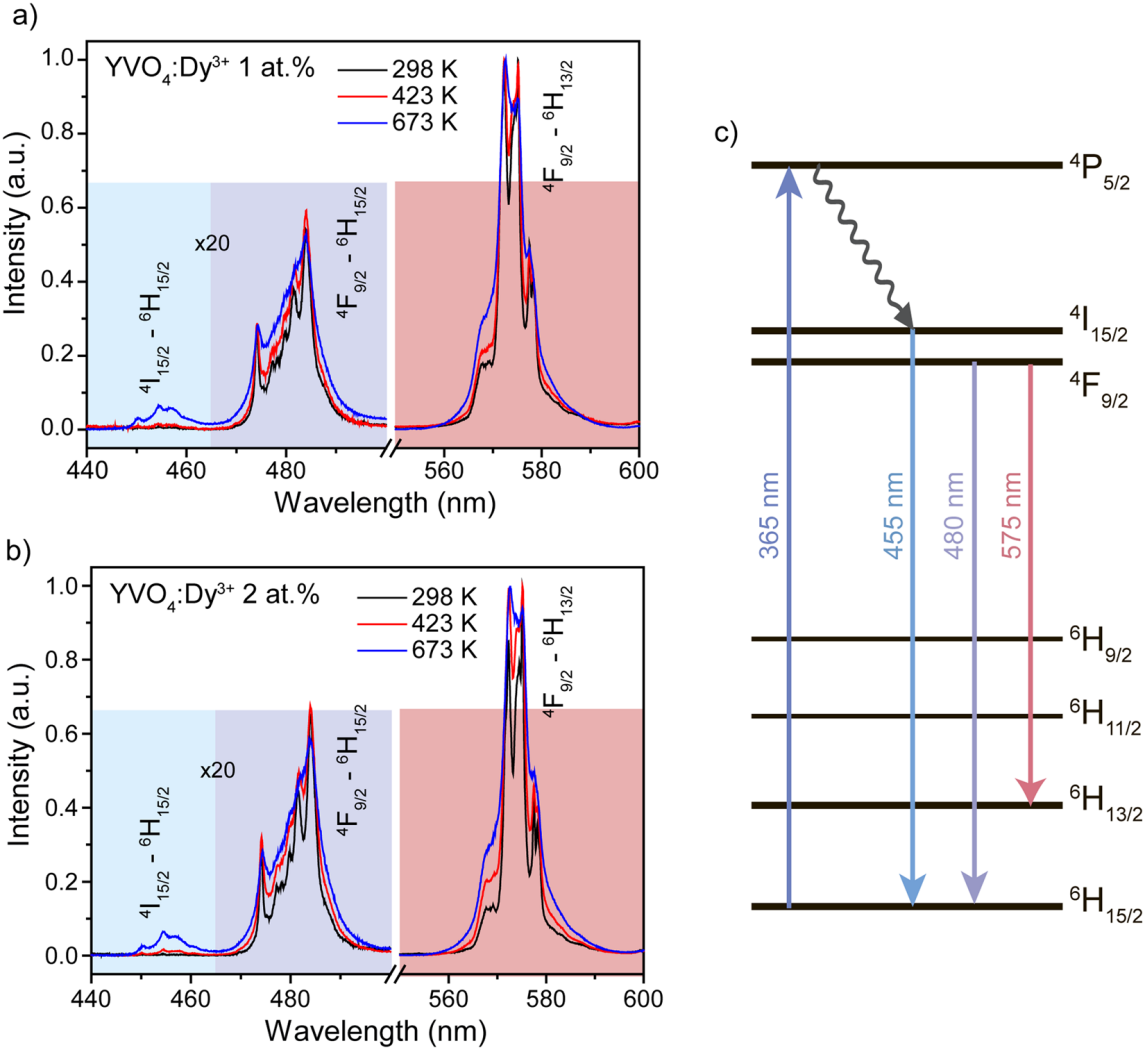
Normalized emission spectra of (**a**) YVO_4_:Dy^3+^ 1 at.% and (**b**) YVO_4_:Dy^3+^ 2 at.% nanopowders at different temperatures. The colored areas are used for the integral intensity ratio calculations. (**c**) Energy levels scheme of YVO_4_:Dy^3+^ nanophosphors.

Figure 12 shows normalized emission spectra of YVO_4_:Dy^3+^ 1 at.% and 2 at.% nanopowders measured at different temperatures (298, 423 and 673 K). The observed emission lines are originated from electron transitions from ^4^I_15/2_ and ^4^F_9/2_ excited states with energy separation of about 1000 cm^−1^ (Fig. 12c). According to definition, ^4^I_15/2_ and ^4^F_9/2_ are thermally coupled levels, therefore ratiometric approach for transitions from these excited states can be utilized to determine local temperature. Luminescence intensity ratio (LIR) between ^4^I_15/2_–^6^H_15/2_ and ^4^F_9/2_–^6^H_15/2_ transitions (R_455/480_), as well as ratio between ^4^I_15/2_–^6^H_15/2_ and ^4^F_9/2_–^6^H_13/2_ transitions (R_455/575_) were used for thermal sensing. It should be noted that calculation of luminescence ratios was based on the integrated intensities of transitions, because such approach gives better accuracy comparing with peak intensities^78^.

**Figure 13:**
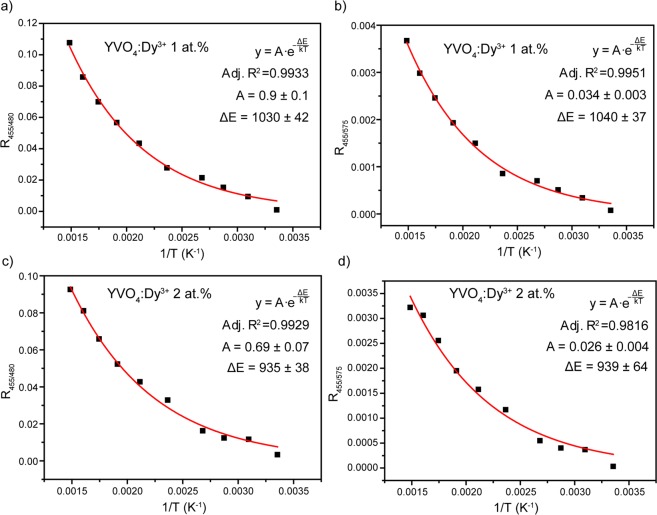
Luminescence intensity ratio (R_455/480_) of (**a**) YVO_4_:Dy^3+^ 1 at.% and (**c**) YVO_4_:Dy^3+^ 2 at.% nanopowders as a function of temperature. Luminescence intensity ratio (R_455/575_) of (**b**) YVO_4_:Dy^3+^ 1 at.% and (**d**) YVO_4_:Dy^3+^ 2 at.% nanopowders as a function of temperature. Red lines correspond to the best fitting with Eq. (5).

The variations of the LIR values of YVO_4_:Dy^3+^ 1 at.% and YVO_4_:Dy^3+^ 2 at.% nanocrystalline powders as a function of the temperature are presented in Fig. 13. Temperature induced change of LIR is caused by electron re-distribution at the energy levels according to the Boltzmann formula:

5 $$R=A\cdot \exp (-\frac{{\rm{\Delta }}E}{kT})$$

where A is a temperature-independent constant, ΔE is the energy gap between thermally coupled energy levels, k is the Boltzmann’s constant and T is the absolute temperature. As can be seen, the experimental data were successfully fitted with eq. (5) and the obtained parameters were presented in the graphs.

To estimate the performance of the thermometer, absolute (S_a_) and relative (S_r_) thermal sensitivities are usually used. The absolute thermal sensitivity shows the absolute LIR change with temperature variation and is defined as follows:

6 $${S}_{a}=\frac{dR}{dT}=R\frac{{\rm{\Delta }}E}{k{T}^{2}}$$

It is obviously from Eq. (6) that S_a_ depends on absolute LIR value, which can be significantly changed by manipulating LIR calculation procedure (for instance, change of integration limit for calculation of integral intensity of emission transition). Therefore, absolute thermal sensitivity cannot be used for the fair comparison among different systems^79^. To compare thermometers irrespective to their nature and sensing parameter, the relative thermal sensitivity is introduced. S_r_ shows normalized change of LIR with temperature variation and is defined as follows:

7 $${S}_{r}=\frac{1}{R}\frac{dR}{dT}=\frac{{\rm{\Delta }}E}{k{T}^{2}}$$

**Figure 14:**
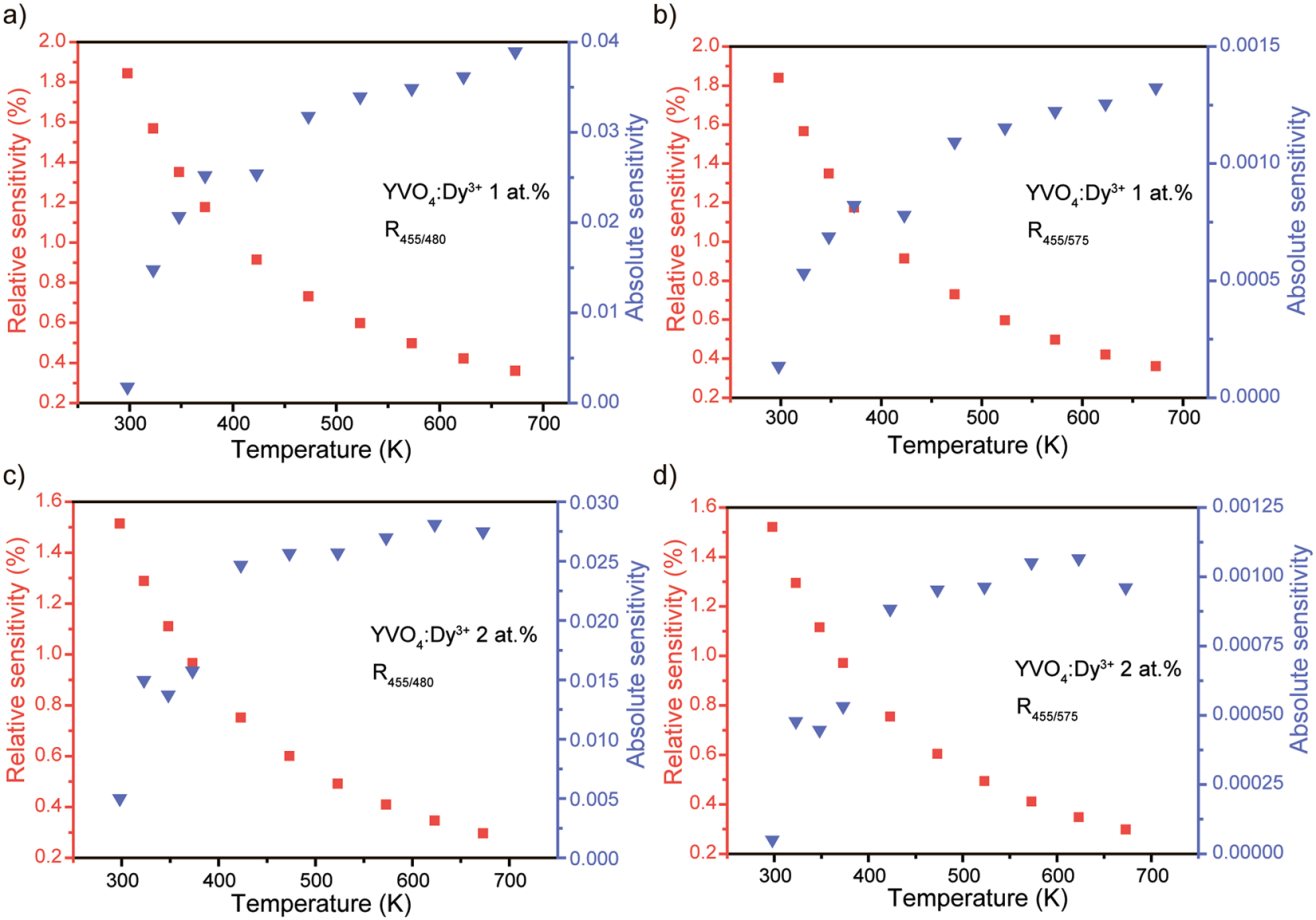
Variation of absolute (S_a_) and relative (S_r_) thermal sensitivity dependence on temperature for (**a**,**b**) YVO_4_:Dy^3+^ 1 at.% and (**c**,**d**) YVO_4_:Dy^3+^ 2 at.%.

The variation of the S_a_ and S_r_ value with temperature from 298 to 673 K for both studied LIRs is presented in the Fig. 14. The observed temperature dependences of S_a_ and S_r_ demonstrate opposite behavior: temperature increase leads to the gradual growth of S_a_ and monotonic decline of S_r_. Temperature dependence in both studied LIRs is originated from the same excited levels: ^4^I_15/2_ and ^4^F_9/2_. So, it is unsurprising that the maximal relative thermal sensitivity (T = 298 K) for R_455/480_ and R_455/575_ ratios is similar: 1.8% K^−1^ (YVO_4_:Dy^3+^ 1 at.%) and 1.5% K^−1^ (YVO_4_:Dy^3+^ 2 at.%). However, the absolute thermal sensitivities differ significantly: 0.039 K^−1^@673 K (R_455/480_) and 0.0013 K^−1^@673 K (R_455/575_) in case of YVO_4_:Dy^3+^ 1 at.%. Noteworthy that increase of Dy^3+^ doping concentration results in worsening of thermal sensitivity.

In comparison with Er^3+^, Tm^3+^ or Nd^3+^ ions, dysprosium ions are relatively rare utilizing for temperature sensing. Among other Dy^3+^ single doped materials, the obtained S_r_ values are comparable to the values reported in the literature: 1.7% K^−1^ for BaYF_5_:Dy^3+^ NPs^80^, 1.6% K^−1^ for Gd_2_Ti_2_O_7_:Dy^3+^ NPs^81^, 1.7% K^−1^ for YAG:Dy^3+^ microcrystals^82^, 1.7% K^−1^ and 1.3% K^−1^ for Dy:Y(acac)_3_ and Dy:Y(acac)_3_(phen) molecular crystals, respectively^83^.

The minimum temperature uncertainty (ΔT) provides information about accuracy of thermal sensing which can be derived using this material. There are several experimental techniques to obtain minimum temperature uncertainty, which were discussed and compared in our previous work^78^. Here, ΔT was estimated from consecutive emission spectra measured at fixed heating stage temperature (Figs S2 and S3). Due to the rather wide temperature sensing region, temperature uncertainty was obtained for 323 and 473 K. The obtained value of ΔT is lying in the range of 2–7 K depending on the measured temperature, used luminescence intensity ratio and doping concentration.

**Figure 15:**
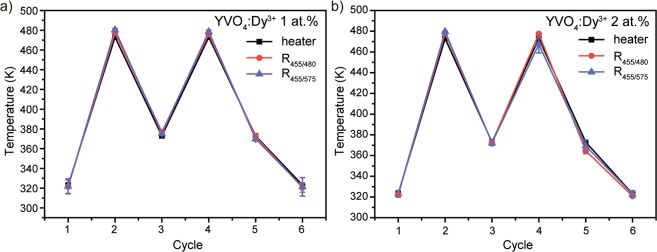
Calculated and measured temperature in heating-cooling cycles for (**a**) YVO_4_:Dy^3+^ 1 at.% and (**b**) YVO_4_:Dy^3+^ 2 at.%.

Third important factor for the assessment of the precision of a thermometric system is repeatability. We tested repeatability of YVO_4_:Dy^3+^ thermometers over cyclic heating-cooling measurements (Fig. 15). During the experiment, we increased and decreased the temperature within the thermal range of 323–473 K. Black squares indicate the actual temperature of heater, whereas red circles and blue triangles present temperature obtained with R_455/480_ and R_455/575_ luminescence intensity ratio, respectively. Taking into account temperature uncertainty, we can conclude good repeatability of considered YVO_4_:Dy^3+^ nanopowders: temperatures obtained using optical thermometry are repeated from cycle to cycle and they are in good agreement with the actual heater temperature.

## Summary

Series of nanocrystalline Dy^3+^-doped YVO_4_ samples with different calcination temperature and doping concentration were prepared by modified Pechini method. XRD study defines that YVO_4_:Dy^3+^ nanoparticles had tetragonal phase without any impurities. Synthesized samples consisted of quite small nanoparticles with size about 50 nm. EDX technique revealed signals from yttrium, vanadium, oxygen, and dysprosium. Narrow width of Raman lines confirmed good homogeneity and crystallinity of synthesized nanoparticles. Excitation spectrum of Dy^3+^-doped YVO_4_ nanophosphors consisted of broad band and several narrow lines assigned to the pump through matrix host and direct pump of Dy^3+^ ion, respectively. Emission spectrum consisted of the characteristic narrow lines attributed to the intra-configurational 4f-4f transitions. The most intensive luminescence band corresponded to the hypersensitive ^4^F_9/2_–^6^H_13/2_ transition. Calcination temperature increase led to the growth of emission intensity due to the improvement of the crystallinity and decrease of the quenchers number. Study of YVO_4_:Dy^3+^ concentration series demonstrated usual concentration quenching effect. The optimal Dy^3+^ doping concentrations of 1 and 2 at.% were determined for different excitation mechanisms (λ_ex_ = 310 and 365 nm, respectively). Concentration quenching in YVO_4_:Dy^3+^ nanocrystalline powders occurred due to dipole–dipole interaction regardless of the excitation mechanism. The calcination temperature variation did not affect ^4^F_9/2_ lifetime, whereas increase of doping concentration resulted in its gradual decline. CIE coordinates of YVO_4_:Dy^3+^ nanoparticles did not depend on either calcination temperature or doping concentration. YVO_4_:Dy^3+^ 1 at.% and YVO_4_:Dy^3+^ 2 at.% nanocrystalline powders were tested as ratiometric luminescence thermometers. Temperature sensing based on R_455/480_ and R_455/575_ luminescence intensity ratios was demonstrated within wide thermal range of 298–673 K. The maximum relative thermal sensitivity was 1.8% K^−1^@298 K, whereas the minimum temperature uncertainty was found to be 2 K. Thermal cycling experiments showed good repeatability of studied thermometer.

## Methods

YVO_4_:Dy^3+^ samples were prepared by modified Pechini method^32,55^. The doping concentration of Dy^3+^ was 0.5, 1, 2, 5, 10, 15 at.% to Y^3+^ in YVO_4_ host. The starting materials were yttrium oxide (Y_2_O_3_), dysprosium oxide (Dy_2_O_3_), vanadium oxide (V_2_O_5_), concentrated nitric acid (HNO_3_), citric acid (C_6_H_8_O_7_) and ethylene glycol (C_2_H_6_O_2_). Y_2_O_3_ and Dy_2_O_3_ were dissolved in concentrated HNO_3_ with heated to form nitrates solution. Then aqueous solution of citric acid was added to yttrium-dysprosium nitrates mixture (with volume ratio 1:1). The chemical reaction is as follows:

$${\rm{Me}}{({{\rm{NO}}}_{3})}_{3}+{{\rm{3C}}}_{6}{{\rm{H}}}_{8}{{\rm{O}}}_{7}=[{\rm{Me}}{({{\rm{C}}}_{6}{{\rm{H}}}_{8}{{\rm{O}}}_{7})}_{3}]{({{\rm{NO}}}_{3})}_{3}$$

Then V_2_O_5_ was dissolved in citric acid to form VO(C_6_H_7_O_7_)_2_ according to the following reaction:

$${{\rm{V}}}_{2}{{\rm{O}}}_{5}+{{\rm{5C}}}_{6}{{\rm{H}}}_{8}{{\rm{O}}}_{7}={\rm{2VO}}{({{\rm{C}}}_{6}{{\rm{H}}}_{7}{{\rm{O}}}_{7})}_{2}+{{\rm{C}}}_{6}{{\rm{H}}}_{8}{{\rm{O}}}_{7}+1/2\,{{\rm{CO}}}_{2}+{{\rm{H}}}_{2}{\rm{O}}$$

After that previous metals citrate complex ([Me(C_6_H_8_O_7_)_3_](NO_3_)_3_) was added with stirring and heated. The green solution was formed. Then ethylene glycol was added to the above mixture (volume ratio with of ethylene glycol and citric acid solution was 1:4 respectively). The formation of citrate transparent gel was observed. The obtained polymer gel was transferred to a crucible and then was placed in a furnace maintained at a temperature of 500 °C/1 h to burn off the organic components. The brown powder was formed. It was grinded in mortar with adding the potassium chloride in weight ratio 1:1. Then powder mixture kept in a muffle furnace maintained at predefined temperature/1.5 h. We used 850, 900, 950, 1000, 1100 and 1200 °C as the second calcination temperature. After the second thermal treatment white powder was removed from the furnace, centrifuged, washed three times with distilled water to remove potassium chloride and, finally, dried. Thus, YVO_4_: Dy^3+^ nanocrystalline powders were synthesized.

X-ray diffraction patterns were registered with the powder diffractometer UltimaIV (Rigaku) in Bregg-Bretano geometry with CuKα1 radiation (λ = 1.54059 Å) in the 2θ range from 7° to 80°. Phase identification was carried out using a powder diffraction database PowderDiffractionFile (PDF-2, 2011). The unit cell parameters were estimated using UnitCell software. Electron micrograph images and elemental analysis were obtained using Zeiss Merlin electron microscope with Oxford Instruments INCAx-act accessory. Raman spectrum was measured on Bruker SENTERRA Raman Microscope with semiconductor laser 488 nm as an excitation source. Steady-state and kinetics photoluminescence properties were studied with modular fluorescence spectrometer Fluorolog-3 (HORIBA Jobin Yvon). All thermal measurements were performed by using T64000 Raman Spectrometer. The Dy^3+^-doped YVO_4_ NPs were optically excited with a 374 nm diode laser Coherent CUBE. The laser beam was focused into the sample by using a 4x long working distance microscope objective (NA 0.1). The fluorescence was collected by using the same microscope objective and was spectrally analyzed by single spectrometer and Peltier cooled Synapse CCD detector. The temperature was controlled with a heating stage controlled with heating stage Linkam TS1000 with 0.1 °C temperature stability and 0.1 °C set point resolution.

## Supplementary information

Supporting Information
