# Supplementary material for: Structural, luminescence and thermometric properties of nanocrystalline YVO4:Dy3+ temperature and concentration series
Source: Sci Rep. 2019 Feb 14;9:2043. doi: 10.1038/s41598-019-38774-6 (PMC6376052; doi:10.1038/s41598-019-38774-6)
Supplement: Supplementary file 1 — Supporting Information [file 41598_2019_38774_MOESM1_ESM.pdf]

Structural, luminescence and thermometric properties of nanocrystalline $YVO_4:Dy^{3+}$ temperature and concentration series

I.E. Kolesnikov[*,1,2], A.A. Kalinichev[1], M.A. Kurochkin[1], E.V. Golyeva[3,4], A.S. Terentyeva[3], E.Yu. Kolesnikov[5], E. Lähderanta[2]

[1] St. Petersburg State University, 7/9 Universitetskaya nab., 199034, St. Petersburg, Russia
[2] Lappeenranta University of Technology LUT, Skinnarilankatu 34, 53850, Lappeenranta, Finland
[3] *Peter the Great St. Petersburg Polytechnic University, St. Petersburg, Russia*
[4] Scientific and Technological Institute of Optical Material Science, VNTs S. I. Vavilov State Optical Institute, Babushkina 36-1, 192171, St. Petersburg, Russia
[5] *Volga State University of Technology, Lenin sqr. 3, 424000, Yoshkar-Ola, Russia*

Calcination temperature and doping concentration effects on the emission color of $YVO_4:Dy^{3+}$ nanophoshors were studied in terms of the Commission Internationale de L’Eclairage (CIE) chromaticity coordinates. The obtained results are presented in Figure S1.

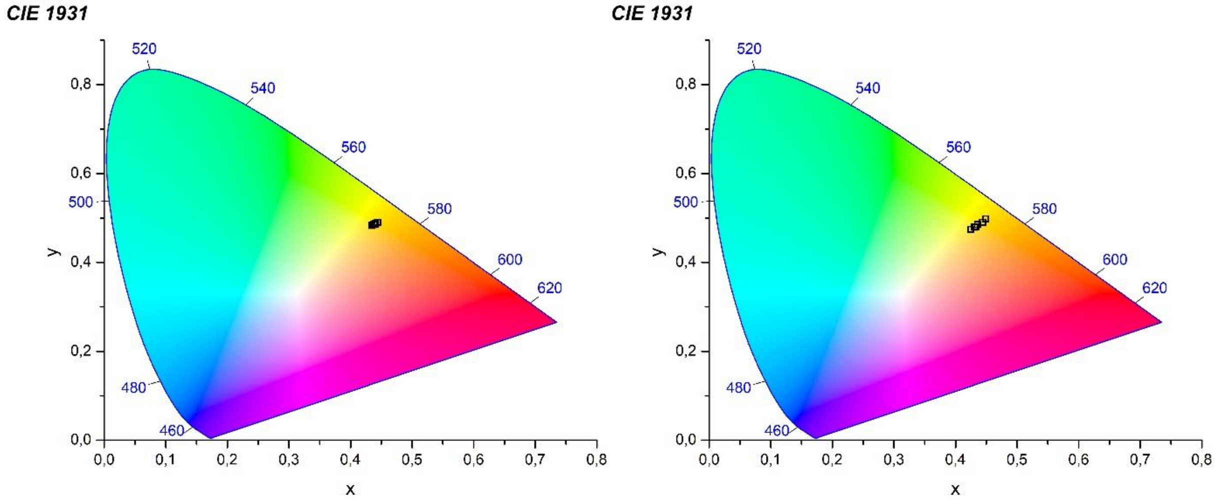

Figure S1. CIE coordinates of $YVO_4:Dy^{3+}$ temperature series (left) and $YVO_4:Dy^{3+}$ concentration series (right).

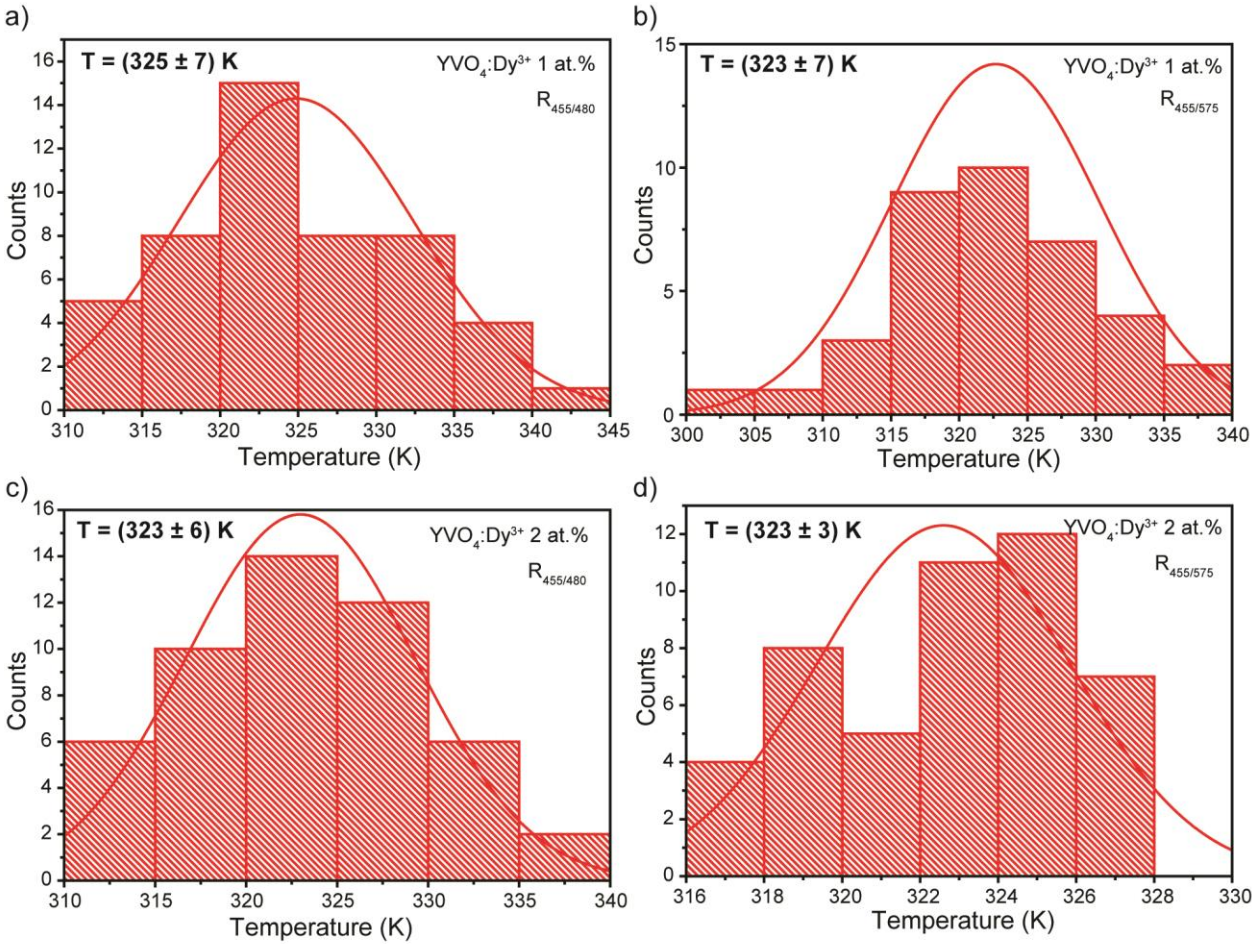

Figure S2. (a) Thermal resolution using $R_{455/480}$ measured for $YVO_4:Dy^{3+}$ 1 at.% at 323 K; **(b)** thermal resolution using $R_{455/575}$ measured for $YVO_4:Dy^{3+}$ 1 at.% at 323 K; **(c)** thermal resolution

using $R_{455/480}$ measured for $YVO_4:Dy^{3+}$ 2 at.% at 323 K; **(d)** thermal resolution using $R_{455/575}$ measured for $YVO_4:Dy^{3+}$ 2 at.% at 323 K.

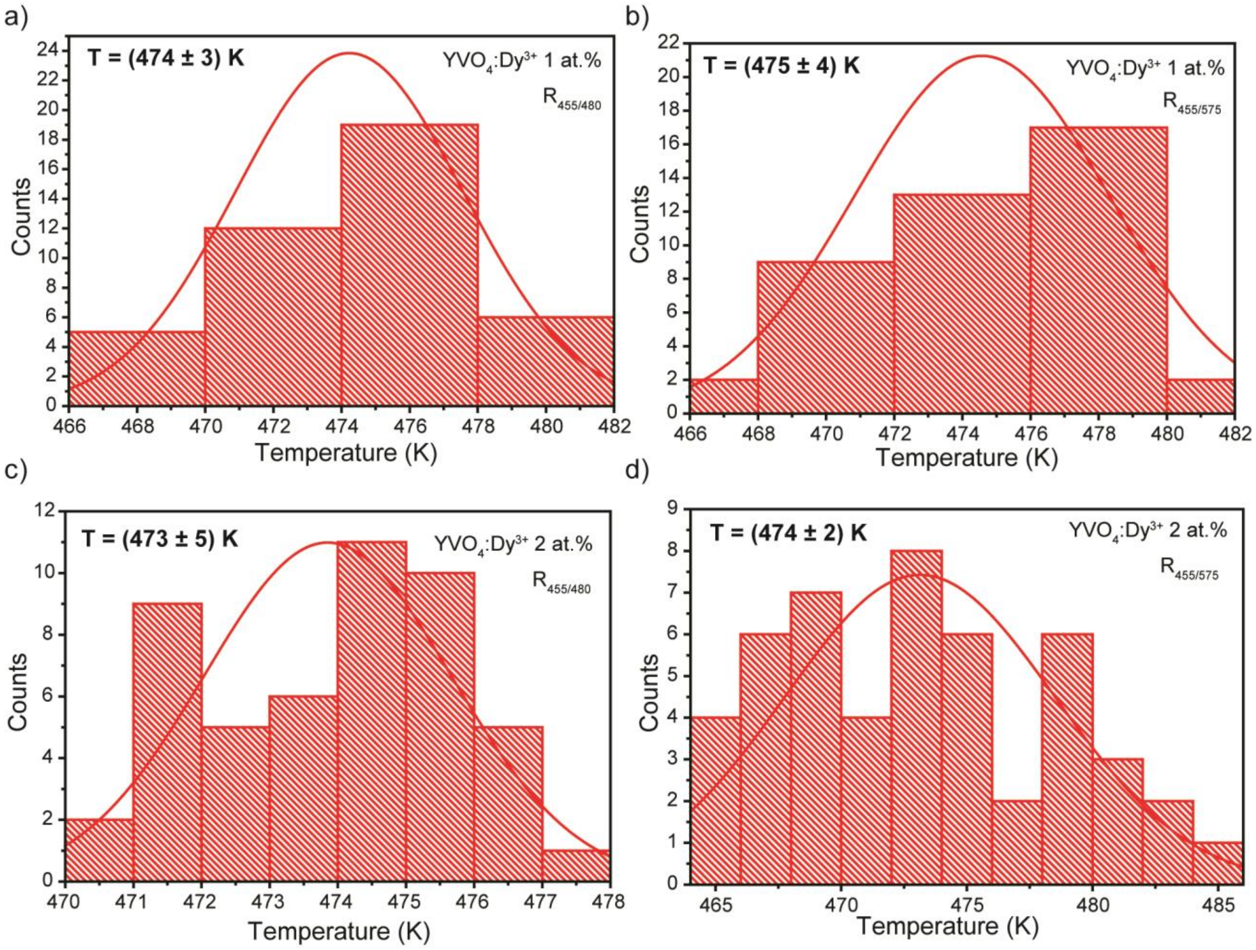

Figure S3. (a) Thermal resolution using $R_{455/480}$ measured for $YVO_4:Dy^{3+}$ 1 at.% at 473 K; **(b)** thermal resolution using $R_{455/575}$ measured for $YVO_4:Dy^{3+}$ 1 at.% at 473 K; **(c)** thermal resolution using $R_{455/480}$ measured for $YVO_4:Dy^{3+}$ 2 at.% at 473 K; **(d)** thermal resolution using $R_{455/575}$ measured for $YVO_4:Dy^{3+}$ 2 at.% at 473 K.
